# FTY720 Story. Its Discovery and the Following Accelerated Development of Sphingosine 1-Phosphate Receptor Agonists as Immunomodulators Based on Reverse Pharmacology

**Published:** 2007-09-06

**Authors:** Kunitomo Adachi, Kenji Chiba

**Affiliations:** 1 Chemistry Laboratory, Pharmaceuticals Research Division, Mitsubishi Pharma Corporation, 1000, Kamoshida-cho, Aoba-ku, Yokohama, 227-0033, Japan; 2 Research Laboratory III (Immunology), Pharmaceuticals Research Division, Mitsubishi Pharma Corporation, 1000, Kamoshida-cho, Aoba-ku, Yokohama, 227-0033, Japan

**Keywords:** FTY720, Fingolimod, sphingosine 1-phosphate, sphingosine 1-phosphate receptor agonist, immunomodulator, multiple sclerosis

## Abstract

Fingolimod (FTY720) is the first of a novel class: sphingosine 1-phosphate (S1P) receptor modulator and is currently in phase 3 clinical trials for multiple sclerosis (MS). FTY720 was first synthesized in 1992 by chemical modification of an immunosuppressive natural product, ISP-I (myriocin). ISP-I was isolated from the culture broth of *Isaria sinclairii*, a type of vegetative wasp that was an ‘eternal youth’ nostrum in traditional Chinese medicine. ISP-I is an amino acid having three successive asymmetric centers and some functionalities. We simplified the structure drastically to find a nonchiral symmetric 2-substitued-2-aminopropane-1,3-diol framework for an *in vivo* immunosuppressive activity (inhibition of rat skin allograft rejection test or prolonging effect on rat skin allograft survival) and finally discovered FTY720. During the course of the lead optimization process, we encountered an unexpected dramatic change of the mechanism of action with an *in vivo* output unchanged. Since it proved that FTY720 did not inhibit serine palmitoyltransferase that is the target enzyme of ISP-I, reverse pharmacological approaches have been preformed to elucidate that FTY720 is mainly phosphorylated by sphingosine kinease 2 *in vivo* and the phosphorylated drug acts as a potent agonist of four of the five G protein coupled receptors for S1P: S1P_1_, S1P_3_, S1P_4_ and S1P_5_. Evidence has accumulated that immunomodulation by FTY720-P is based on agonism at the S1P_1_ receptor. Medicinal chemistry targeting S1P_1_ receptor agonists is currently in progress. The FTY720 story provides a methodology where *in vivo* screens rather than *in vitro* screens play important roles in the lead optimization. Unlike recent drug discovery methodologies, such a strategy as adopted by the FTY720 program would more likely meet serendipity.

## Introduction

**Figure 1.**
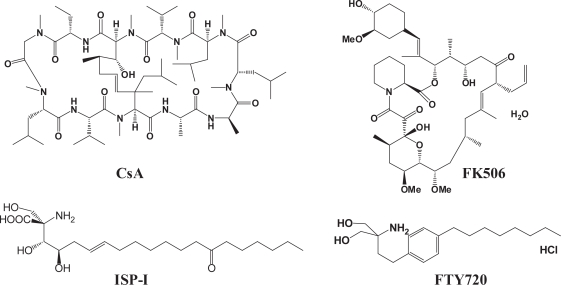
Structure of CsA, FK506, ISP-I and FTY720.

Immunosuppressants are clinically important for organ transplantations and the treatment of autoimmune diseases such as rheumatoid arthritis (RA), multiple sclerosis (MS), systemic lupus erythematosus (SLE), and psoriasis. Calcineurin inhibitors cyclosporin A (CsA) and tacrolimus (FK506) are clinically used as an immunosuppressant (Fig. 1). (1, 2) Both CsA and FK506 inhibit the production of type 1 helper T cell-derived cytokines such as interleukin 2 (IL-2) and interferon-gamma that induce cytotoxic T cells but induce renal dysfunction and other side effects at higher doses. (3, 4) CsA was isolated from a fungus, *Tolypocladium inflatum*. The historic discovery of the immunosuppressant from a fungus stimulated other researchers to search for immunosuppressants from various fungi. Fujita and coworkers also screened other fungi as a part of their screening program of fungal extracts and finally isolated an immunosuppressant, ISP-I that was 10- to 100-fold more potent than CsA from *Isaria sinclairii*, a kind of vegetative wasp that is an “eternal youth” nostrum in traditional Chinese herbal medicine (Fig. 1). (5) ISP-I was found to inhibit mouse allogeneic mixed lymphocyte reaction (MLR) *in vitro* and to be effective in rat skin allograft *in vivo*. (6, 7) The chemical structure of ISP-I since proved to be identical to those of myriocin and thermozymocidine, which had been isolated as an antifungal agent from *Myriococcum albomyces* (ATCC16425) and *Mycelia sterilia* (ATCC20349), respectively. (8, 9, 10)

ISP-I is structurally even simpler than CsA and FK506. While the molecular weight was rather diminished compared with CsA, ISP-I was toxic and had unfavorable physicochemical properties such as low solubility. Lead optimization of ISP-I was based on the simplification of the structure. Eventually, the modification led to a novel synthetic compound, 2-amino-2-[2-(4-octylphenyl)ethyl] propane-1,3-diol hydrochloride (FTY720), which has more potent immunosuppressive activity and less toxicity than the lead ISP-I (Fig. 1). (11, 12)

MS is a progressive and debilitating disorder of the central nervous system that frequently affects young adults. MS is believed to be one of autoimmune diseases in which autoreactive T cells attack myelin sheaths, leading to demyelination and axonal damage. (13) Interferon beta and glatiramer acetate are currently approved as immunomodulating treatments for MS. Both are administered either subcutaneously or intramuscularly. Interferons are associated with systemic reactions in more than 60% of patients, with implications for adherence to treatment. Orally available low molecular drugs with less toxicity have been longed for. In a proof-of-concept study (Phase 2 clinical trial), FTY720 reduced the number of lesions detected on magnetic resonance imaging (MRI) and clinical disease activity in patients with relapsing multiple sclerosis. (13) In this study, FTY720 was well tolerated. While the treatment induced a transient reduction of heart rate that was maximal at six hours after the first dosing, the heart rate returned to baseline with continuous treatment. It was shown that oral FTY720 may be a treatment option for MS. International Phase 3 clinical trials for further evaluation in large-scale, long-term clinical studies are currently under way.

Applications of FTY720 for other indications have been widely investigated. It has been reported that FTY720 would be a potent anticancer agent. (14–17) It has been reported that an inhibitory effect of FTY720 on airway inflammation has been reported, suggesting that it may be useful for allergic diseases such as asthma. (18) It has been suggested that FTY720 may be efficacious in beta-amyloid-related inflammatory diseases such as Alzheimer’s disease. (19) A protective effect against immune liver injury models has also been reported. (20)

## A Fortuitous Discovery of FTY720

Naturally occurring ISP-I showed a potent inhibitory effect on mouse allogeneic mixed lymphocyte reaction (MLR) *in vitro* and prolonged rat skin graft survival time *in vivo*. (6) Optimization was conducted using both *in vitro* and *in vitro* assays as screens. The *in vitro* assay is based on the principle that mouse MLR is caused by T cell proliferation stimulated with alloanitigen when mouse lymphocytes from two different strains are co-cultured. We carried out the assay by culturing BALB/c mouse spleen cells as responder cells with mitomycin-pretreated C57BL/6 mouse spleen cells as stimulator cells. It should be mentioned that the selection of this particular evaluation system as the *in vitro* screen became crucial in the latter half of the FTY720 story because it turned out some time after the discovery of FTY720 that different assay systems using mice other than the above-mentioned ones did not work in evaluating *in vitro* immunosuppressive activity of FTY720-related compounds. Test compounds were evaluated in skin allograft in major histocompatibility complex-compatible rat strain combination between LEW donor and F344 recipient *in vivo*. Importantly, all the compounds synthesized were basically evaluated using the whole animal model (rat skin allograft model). It should be emphasized that FTY720 program was sustained mainly by the highly reliable *in vivo* screening system that required proficient transplant operation skills and long evaluation periods (sometimes more than fifty days).

**Figure 2.**
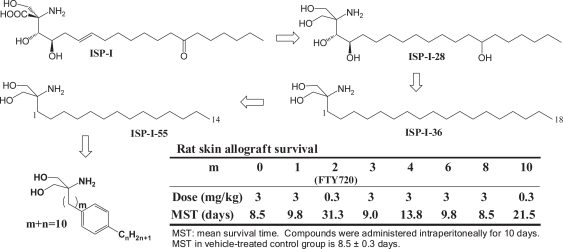
Optimization of the structure starting from ISP-I.

Immunosuppressive ISP-I homologues, mycestericins were isolated from another fungus. (7, 21, 22) The SAR studies on these ISP-I homologues, including semi-synthetic derivatives, revealed that neither the functionalities (the 14-ketone, the 6-double bond, and the 4-hydroxy group) nor the configuration at the carbon bearing the 3-hydroxy group are so important for its activity; however, critical problems such as toxicity and insolubility remained to be solved. Therefore, simplification of the structure of ISP-I was conducted to reduce its toxicity and to improve its physicochemical properties (Fig. 2). The simplification process consisted of removal of the side chain functionalities and elimination of chiral centers. During the course of the process, a compound (ISP-I-28) where the carboxylic acid of ISP-I was transformed to the hydroxymethyl group was found not only to prolong the rat skin survival time more effectively but also to be approximately 30-fold less toxic than ISP-I. (7) Since the conversion of the carboxylic acid to the hydroxymethyl group proved to be highly advantageous from toxicological (less toxic), pharmacological (more effective), synthetic (reduction of the number of asymmetric carbons), and physicochemical (better solubility) points of view, a thorough simplification was conducted to finally lead to much more simplified structure, 2-alkyl-2-aminopropane-1,3-diol represented by an analog having an eighteen carbon alkyl chain, ISP-I-36. The modification of the side chain length led to ISP-I-55. The compound with a fourteen carbon alkyl chain was much more effective both *in vitro* and *in vivo* and much less toxic than ISP-I. (6, 7)

ISP-I-55 was further modified by introducing a phenylene moiety in a proper position within the side chain in the expectation for a favorable conformation restricting effect of the phenyl group and finally FTY720 was synthesized in 1992 (Fig. 2). (11) The position of the phenyl group is highly critical for its potent activity. The other analogs having the phenylene moiety in a different position displayed obviously diminished activity compared to FTY720 (Fig. 2). FTY720 was more potent than ISP-I-55 *in vivo* while it displayed comparable activity to ISP-I-55 *in vitro*. Furthermore FTY720, having a phenyl ring in it, was considered to be more favorable in various tests in ADME and toxicology studies as well as in clinical trials because FTY720 is easier to detect than ISP-I-55 having no aromatic rings. Starting from ISP-I, drastic simplification of the structure was effective in improving *in vitro* and *in vivo* activity, toxicity, and physicochemical properties, leading to the discovery of FTY720 as an immunomodulator.

In 1995, the lead ISP-I was found to inhibit serine palmitoyltransferase (SPT) involved in sphingolipid biosynthesis. (23) It was surprising that neither FTY720 nor ISP-I-55 displayed the inhibitory activity against the enzyme.(12, 24) It is obvious that the mechanism of action changed sometime during the optimization process from ISP-I to FTY720. However, for some time after the discovery of FTY720, the change of the mechanism had not been realized. If the SPT inhibition assay had been substituted for the MLR assay as the first screen, FTY720 might not have been discovered. Fortunately, the target of ISP-I was not known at the time of the FTY720 screening. It was fortuitous that the *in vitro* cell-based evaluating system (allogeneic MLR using the particular combination of BALB/c responder cells and C57BL/6 stimulator cells) worked even after the mechanism of action changed.

Where did the mechanism of action change? Although it remains to be examined, we speculate that it happened at ISP-1-28, ISP-I-36 or somewhere from ISP-1-28 to ISP-I-36. The next question arose as to what is the real mechanism of action of FTY720. Another seven years were needed until the molecular basis of immunosuppressive activity of FTY720 was proposed in 2002.

## FTY720: Pharmacological Actions and Mechanisms of Action

**Figure 3.**
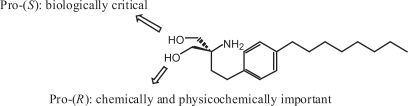
Role of the hydroxymethyl groups of FTY720.

FTY720 contains a prochiral quarternary carbon atom bearing two hydroxylmethyl groups (Fig. 3). Replacement of one of the hydroxylmethyl groups by an alkyl substituent such as methyl afforded an FTY720 analog as a mixture of racemates. Optical resolution of the racemate was performed and pharmacological evaluation of each enantiomer revealed that the pro-(*S*) hydroxymethyl group of FTY720 is much more important for biological activity than the other. (25) While the pro-(*S*) hydroxymethyl group is biologically critical, the biologically less important pro-(*R*) hydroxymethyl group may play important roles chemically and physicochemically in simplifying the molecule (deletion of the chiral center by symmetrization) and improving the solubility.

FTY720, at 0.1 mg/kg or higher doses, significantly prolongs skin or cardiac allograft survival and host survival in lethal graft versus host reaction (GvHR) in rats (Table 1). (26–29) In addition, combination treatment with FTY720 and a subtherapeutic dose of cyclosporin A (CsA) or tacrolimus (FK506) results in a synergistic effect on canine renal allograft as well as rat skin or cardiac allografts. (26, 27, 29–35)

A striking feature of FTY720 is the induction of a marked decrease in the number of peripheral blood lymphocytes, especially T-cells, at doses that prolong allograft survival. (26, 27, 36) Since a whole animal study using this phenomenon was reported, (12, 25) assay systems where the decrease in the number of peripheral blood lymphocytes is counted as an index of immunosuppression has been widely used to evaluate the immunosuppressive activity of test compounds. FTY720 does not impair lymphocyte function, including T-cell activation, but instead induces the sequestration of circulating mature lymphocytes into the secondary lymphoid tissues and decreases T-cell infiltration into grafted organs and this is presumed to be the main mechanism of FTY720 immunosuppressive activity. (34–37, 38) Comparison of the effects of FTY720 in various models including transplantation models and autoimmune models with those of calcinuerin inhibitors has been reported. (29, 39)

**Figure 4.**
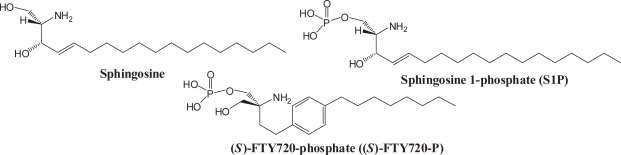
Structures of sphingosine, S1P and (*S*)-FTY720-P.

As described above, FTY720, unlike ISP-I, does not inhibit serine palmitoyltransferase, (12, 24) the first enzyme in sphingolipid biosynthesis, but both molecules are structurally similar to sphingosine. Sphingosine is mainly phosphorylated by sphingosine kinase 1 into S1P that is a multi-functional lysophospholipid mediator and stimulates multiple signaling pathways. S1P binds with nanomolar (nM) affinities to five related G-protein-coupled receptors (GPCRs), termed S1P_1–5_ (formerly Edg-1, -5, -3, -6, and-8). FTY720 is effectively phosphorylated by sphingosine kinase 2 and FTY720-phosphate (FTY720-P) is a high affinity agonist for sphingosine 1-phosphate (S1P) receptors (Fig. 4). (40, 41, 42)

**Figure 5.**
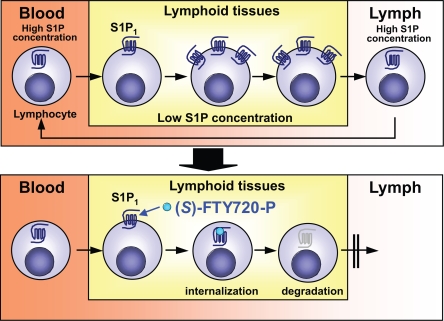
(*S*)-FTY720-P inhibits S1P/S1P_1_-dependent lymphocyte egress from lymphoid tissues by long-term internalization and degradation of S1P_1_.

Evidence has accumulated that S1P receptor type 1 (S1P_1_) is essential for lymphocyte recirculation and that S1P_1_ regulates lymphocyte egress from thymus and secondary lymphoid tissues. (43, 44, 45) In mice whose hematopoietic cells lack S1P_1_, no T-cells are found in the periphery because mature T-cells are unable to exit the thymus and secondary lymphoid tissues. (44) Moreover, S1P_1_-dependent chemotactic responsiveness is strongly up-regulated in T-cell development before exit from the thymus, whereas S1P_1_ is down-regulated during peripheral lymphocyte activation, and this is associated with retention in lymphoid tissues. (44, 46) It has been suggested that FTY720-P internalizes S1P_1_ on lymphocytes and inhibits S1P/S1P_1_-dependent lymphocyte egress from secondary lymphoid tissues and thymus (Fig. 5). (44, 45) FTY720 treatment down-regulates S1P_1_, creating a temporary pharmacological S1P_1_-null state in lymphocytes, (44) providing an explanation for the mechanism of FTY720-induced lymphocyte sequestration; therefore, FTY720-P is termed a functional antagonist. (47) Moreover, it has been reported that S1P induces S1P_1_ internalization and recycling while FTY720-P induces S1P_1_ internalization and degradation.(48) This is a decisive difference between S1P and FTY720-P.

Interestingly, it has been reported that an S1P_1_ antagonist does not affect the number of constitutive blood lymphocytes.(48) It has been proposed that FTY720 may inhibit lymphocyte egress from lymph node (LN) by two independent mechanisms. One involves a down-modulation of S1P_1_ on T cells and a reduced response of cells to the egress signal S1P. The other involves a persistent signaling at S1P_1_ on the sinus lining endothelium in the LN to increase barrier function and reduce transmigration of lymphocytes. (43, 49, 50) Thus, FTY720 may differentially regulate S1P_1_ surface expression in T cells (functional antagonist) and endothelium (agonism). This may be the reason the S1P_1_ receptor antagonist cannot reproduce the action of FTY720. Reverse pharmacology and medicinal chemistry have succeeded in providing an insight into the mechanism of action of FTY720 and have opened an avenue to the development of new S1P receptor agonists.

In terms of the immunosuppressive mechanism of FTY720, several mechanisms other than S1P_1_ receptor agonism of FTY720-P have been proposed. One of those is related to immunosuppressive effects of FTY720-P through the S1P_4_ receptor.^51^ Direct effects of FTY720 itself (not FTY720-P) have also been reported. FTY720 has been reported to inhibit sphingosine lyase (SPL) that is responsible for the degradation of S1P. (52) It has been strongly suggested that SPL is a next promising target for the development of immunomodulators because its inhibition can produce the decease of the number of peripheral lymphocytes like FTY720 treatment. (50, 53) More recently FTY720 has been demonstrated to inhibit cytosolic phospholipase A2 independently of S1P receptors. (54)

## Exploration of Synthetic Methods for FTY720 and FTY720-P

**Figure 6.**
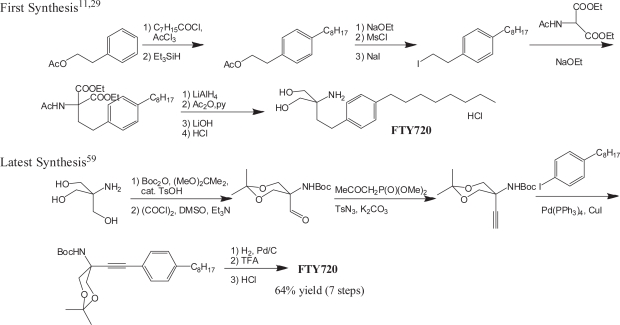
Synthetic routes for FTY720.

Since the first synthetic route for FTY720 was reported, (11, 29) alternative methods have emerged. A shorter synthesis with Friedel-Crafts acylation of octylbenzene as a key step has been reported. (55) A regioselective ring opening of an epoxide with a system of MgSO_4_/MeOH/NaNO_2_ has been applied to synthesis of FTY720. (56) An iron-catalyzed cross-coupling reaction has been used for a practical and scaleable synthesis of the intermediate, octylbenzene. (57) A synthetic route using the Petasis reaction as the key step has been reported. (58) A highly efficient and practical synthesis using Sonogashira coupling reaction as a key step has been reported. (59) The synthetic route gave FTY720 in seven steps and high overall yield (64%) from a commercially available inexpensive material (Fig. 6).

**Figure 7.**
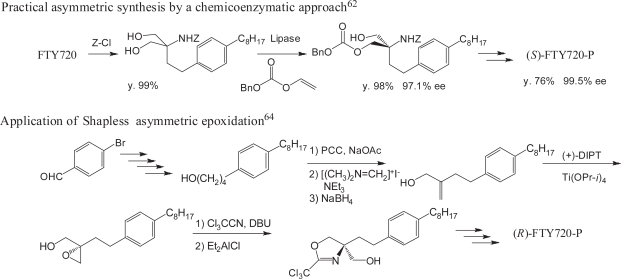
Asymmetric syntheses of FTY720-P enantiomers.

FTY720 is a prochiral symmetric molecule having two hydroxymethyl groups at the quarternary carbon; therefore, purely chemical mono-phosphorylation gives a racemic mixture of (*S*)- and (*R*)-FTY720-P. Several methods for racemic and enantiomerically pure FTY720 phosphate (FTY720-P) have been developed. An efficient direct mono-phosphorylation method using silver(I) oxide, tetrabenzyl pyrophosphate (TBPP), and tetrahexylammonium iodide for racemic FTY720-P has been reported.(60) A properly protected racemic intermediate for FTY720-P were separated using a chiral HPLC column and the absolute configurations of both enantiomers were determined by synthesizing the optically active intermediate starting from an L-serine-derived oxazolizine.(61) A practical asymmetric synthesis of (*S*)- and (*R*)-FTY720-P using lipases and the determination of absolute configurations of FTY720-P have been reported. Only (*S*)-enantiomer was found to be biologically active (Fig. 7).(62)

The absolute configuration of FTY720-P has been determined based on the crystal structure of a protected intermediate and it has been demonstrated that *in vitro* phosphorylation of FTY720 in rats and humans results exclusively in the biologically active (*S*)-enantiomer. (63) A method for the preparation of both enantiomers of FTY720-P from 4-bromobenzaldehyde using the asymmetric Sharpless epoxidation as a key step has also been reported (Fig. 7). (64)

## Development of S1P Agonists Based on Reverse Pharmacology

S1P is known to regulate heart rate, coronary artery blood flow, blood pressure and endothelial integrity. Most recently S1P has been shown to regulate the recirculation of lymphocytes through its high affinity G-protein coupled receptors. FTY720 is mainly phosphorylated *in vivo* by sphingosine kinase 2 to mono phosphate (FTY720-P), which is an agonist of S1P_1, 3, 4, 5_ receptors but not S1P_2_. (40, 41) While FTY720 was well tolerated in clinical studies, it has been reported to produce a transient reduction of heart rate. Researchers have been prompted to elucidate the contribution of individual receptors and solve questions as to which receptor(s) is responsible for immunosuppressive activity of FTY720-P and which receptor(s) causes adverse effects including the transient bradycardia. While S1P_2_- or S1P_3_-kockout mice were successfully obtained, S1P_1_ deletion resulted in the embryonic lethality. (65) Medicinal chemistry not only has helped reverse pharmacology by providing various molecular tools including selective agonists and/or antagonists to unveil the individual functions of S1P receptors but also has been targeting the creation of new S1P receptor agonists as immunosuppressants with fewer adverse effects.

**Figure 8.**
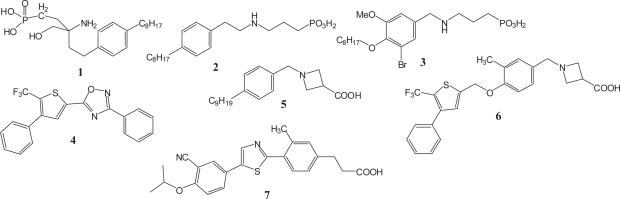
Agents that do not need phosphorylation by sphingosine kinase.

FTY720 and FTY720-P are in equilibrium with each other *in vivo*. In order to directly investigate the pharmacological effects of the agonism of S1P receptors, a nonhydrolyzable analogue of FTY720-P was synthesized as a phosphonate **1**(Fig. 8), which proved successful in eliciting the lymphopenic response although higher doses were required because of lower affinity for S1P receptors as compared to FTY720-P. (66) There are two strategies for exploring S1P receptor agonists as immunosuppressants: one targets prodrugs like FTY720, and the other targets drugs with direct agonism at S1P receptors like FTY720-P and **1**. Researchers in the Merck group have been making efforts based on the latter strategy. Their medicinal chemistry studies were initiated to obtain analogs with enhanced potency *in vitro* and *in vivo* starting from **1**.

A phosphonate analog **2** obtained by the transposition of the 4-alkylphenylethyl group of **1** from the carbon onto the amino group and the deletion of the hydroxymethyl group maintains the nonselective agonism for S1P receptors although the activity decreased (Fig. 8). (67) A further modification gave a first selective compound **3** that selects against the S1P_3_ subtype and shows an attenuated potential to cause the acute bradycardia.(68) This is consistent with the fact that the bradycardia induced by S1P receptor nonselective agonists in wild type mice is abolished in S1P_3_−/− mice. (65, 69) It was found that no T cells were present in the periphery in lymphocyte cell-specific S1P_1_ knockout mice. The phenomenon is similar to that observed in FTY720 treated wild-type mice. It was also found that FTY720 treatment down-regulated S1P_1_ to create a temporary pharmacological S1P_1_-null state in lymphocytes. These findings strongly suggest that S1P_1_ is crucial for controlling the lymphocyte trafficking and that the immunosuppressive activity of FTY720 is based on agonism-driven antagonism at the S1P_1_ receptor. (44) A high throughput screening of Gi-coupled S1P_1_ receptor agonists was conducted over the Merck sample collection, leading to the successful identification of agonists represented by compound **4** having a totally different structure compared to those of sphingosine like agonists synthesized in the earlier stage. **4** was found to be a moderately potent but highly selective S1P_1_ receptor agonist.(70) This orally active S1P_1_ selective agonist has been widely used to elucidate the function of the S1P_1_ receptor and it has been reported that **4** indeed does not induce bradycardia. (65) Here, the goal of medicinal chemistry has been made clear. Compounds having S1P_1_ receptor agonism without S1P_3_ receptor agonism are considered to be desirable S1P receptor immunomodulators with fewer adverse effects such as bradycardia.

Incorporation of the phenyl thiophene moiety of **4** into azetizine carboxylate **5** which had been synthesized as a conformationally constrained analog based on **2** and proved to be a first orally active compound in this series and some modification gave **6** (Fig. 8). **6** and its analogs were found to be about 500- to 1000-fold more selective for S1P_1_ over S1P_3_. (70) Optimization focused especially on PK profiles such as short half-life has been continuously performed on these classes of compounds.(71–76) During the course of the optimization, highly selective and potent S1P_1_ receptor agonists such as **7** were discovered. (75) 7 shows greater than 600-fold selectivity for the S1P_1_ receptor over the other receptors (S1P_2, 3, 4, 5_) and oral administration of **7** to mice drives the lymphocyte-lowering response, which again proved that selective agonism for the S1P_1_ receptor is sufficient for lowering the number of peripheral lymphocytes.

**Figure 9.**
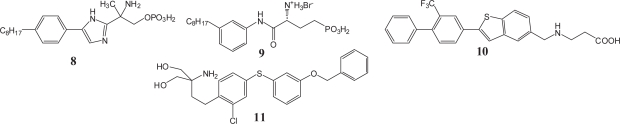
Other S1P receptor agonists/ antagonists.

Other groups also have made synthetic efforts to provide pharmacological tools to understand the individual S1P receptors. Researchers of University of Virginia have been investigating S1P receptor agonists as well as S1P receptor antagonists. (77–82) So far, they have synthesized several compounds exemplified by compound **8** as a modestly selective agonist at the S1P_1_ receptor (79) and compound **9** as an antagonist at the S1P_1_ and S1P_3_ receptors (Fig. 9).(82) Interestingly, no significant change in circulating lymphocyte numbers was observed at any dose of the S1P_1_ antagonist **9**,(79) providing another proof that antagonism at the S1P_1_ receptor is insufficient for mimicking the immunosuppressive action of FTY720.

A pharmacophore based drug design for S1P_3_ receptor antagonists has been reported. (83, 84) An aminocarboxylate analog **10**, a low nanomolar, monoselective agonist of the S1P_1_ receptor has been reported to be effective in an animal model of transplantation (Fig. 9). (85) Selective agonists for the S1P_4_ and S1P_5_ receptors have been developed.(86) These selective agents are expected to be molecular tools to explore the pharmacological roles of the S1P receptors. Recently a prodrug type (FTY720 type) S1P receptor agonist, KRP-203 (**11**) has been reported. (87, 88) KRP-203 is structurally similar to FTY720 but has a lower potential to induce heart rate reduction in guinea pigs probably because KRP-203 phosphate selectively activates the S1P_1_ receptor but not the S1P_3_ receptor. It should be emphasized that the appearance of **11** indicates that the exploration of FTY720 analogs keeping its basic 2-amonopropane-1,3-diol skeleton could lead to selective agonists for the S1P_1_ receptor. Exploration of second-generation S1P receptor agonists will continue on the basis of both strategies.

It would be very natural that a question is raised as to why FTY720 is effective in CNS diseases such as MS while the discussions on its mechanism have been focused mainly on the peripheral events such as the peripheral lymphocyte number decreasing. A key to solve this problem has been provided, suggesting that FTY720 easily crosses the blood-brain barrier (BBB) and its therapeutic effects on MS may be displayed partly through the S1P_5_ receptor in the CNS. (50) Medicinal chemistry will contribute toward answering the issue by providing such chemical tools that resemble FTY720 but do not cross the BBB. Such chemical tools would also be useful for examining whether or not highly selective agonists for the S1P_1_ receptor are as effective in MS in humans as FTY720 one of whose metabolite, FTY720-P is a relatively non-selective S1P receptor agonist.

## Conclusion

In summary, FTY720, after enantiospecific mono-phosphorylation, acts as an agonist at the S1P_1_ receptor, down-regulates and degrades the S1P_1_ receptor on lymphocytes, and inhibits lymphocyte egress from secondary lymphoid tissues and thymus. FTY720 causes the sequestration of circulating mature lymphocytes into lymphoid tissues and modulates the recirculation of lymphocytes between blood and lymphoid tissues. Consequently, it is presumed that FTY720 decreases the trafficking and the infiltration of antigen-specific T-cells into grafted organs or inflammatory sites in autoimmune diseases, thereby exerting powerful immunosuppressive activity. Since FTY720 possesses a completely new mechanism of action, FTY720 should be a useful tool for the prevention of transplant rejection and a new therapeutic approach for autoimmune diseases, including MS, RA, and SLE.

About fifteen years have passed since FTY720 was first synthesized in 1992. FTY720 showed excellent *in vivo* immunosuppressive activities in skin and cardiac allograft models and various autoimmune disease models (89) although the molecular mechanism underlying the pharmacological effects was not identified. That stimulated reverse pharmacology to explore FTY720’s molecular mechanism. Since it was elucidated that FTY720’s target is the S1P receptors in 2002, very rapid explorations for understandings of S1P receptors and their signaling have been developed. It has also been elucidated that S1P induces S1P_1_ internalization and recycling while FTY720-P induces S1P_1_ internalization and degradation, which is a decisive difference between S1P and FTY720-P. Moreover, the development of second generations of FTY720 follow-up agents has been accelerated. FTY720 has been making enormous impacts on various research fields.

In this review we described the serendipitous discovery of FTY720 starting from a naturally occurring immunosuppressive agent ISP-I. In our FTY720 program, the drug discovery stage was driven mainly by a whole animal assay using a rat skin allograft model *in vivo*. It was fortunate for us that the mechanism of the lead ISP-I was elucidated after the discovery of FTY720. Although we were unaware of the change of mechanism from SPT inhibition to S1P receptor agonism, the *in vitro* cell-based assay continued to work. The FTY720 story reminds us that old-fashioned strategies using whole animal assays as the major screens are still highly useful for the discovery of drugs with totally new mechanisms of action.

## Figures and Tables

**Table 1.** Pharmacological properties of FTY720 and calcineurin inhibitors (CsA and FK506).

**Response suppressed**	**Species**	**FTY720**	**CsA**	**FK506**
Allograft rejection	Rat (skin)	0.1 mg/kg	3 mg/kg	0.3 mg/kg
	Rat (heart)	0.1 mg/kg	3 mg/kg	0.3 mg/kg
Combination with CsA	Rat (skin)	0.1 mg/kg		
	Rat (heart)	0.1 mg/kg		
	Dog (Kidney)	0.03 mg/kg		
	Monkey (Kidney)	0.1 mg/kg		
GvHR	Rat	0.1 mg/kg	3 mg/kg	1 mg/kg
DTH (MeHSA)	Mouse	0.03 mg/kg	3 mg/kg	0.03 mg/kg
Antibody production (to SRBC)	Rat	0.1 mg/kg	3 mg/kg	0.03 mg/kg
Adjuvant-induced arthritis	Rat	0.1 mg/kg	3 mg/kg	1 mg/kg
Collagen-induce arthritis	Rat	0.1 mg/kg	3 mg/kg	1 mg/kg
EAE	Rat	0.1 mg/kg	10 mg/kg	1 mg/kg
Lupus nephritis (MRL/lpr)	Rat	0.1 mg/kg	10 mg/kg	1 mg/kg
Lymphopenia	Mouse	0.1 mg/kg		
	Mouse	0.1 mg/kg		
	Rat	0.1 mg/kg		
	Dog	0.03 mg/kg		
	Monkey	0.1 mg/kg		

FTY720: Fingolimod; CsA: cyclosporin A; FK506:tacrolimus; GvHR, graft versus host reaction; DTH: delayed-type hyper sensitivity;MeHA S,methylated human serum albmin; SRBC, sheep red blood cells; EAE, experimental autoimmune encephalomyelitis.
